# Differential circulating cytokine profiles in acute coronary syndrome versus stable coronary artery disease

**DOI:** 10.1038/s41598-024-68333-7

**Published:** 2024-07-27

**Authors:** Eveliina Maaniitty, Juho Jalkanen, Sami Sinisilta, Jarmo Gunn, Tuija Vasankari, Fausto Biancari, Sirpa Jalkanen, K. E. Juhani Airaksinen, Maija Hollmen, Tuomas Kiviniemi

**Affiliations:** 1 Heart Center, Turku University Hospital and University of Turku, POB 52, 20521 Turku, Finland; 2 Vascular Surgery, Turku University Hospital and University of Turku, POB 52, 20521 Turku, Finland; 3 Medicity Research Laboratory, University of Turku, Tykistökatu 6A, 20520 Turku, Finland; 4 Department of Medicine, South Karelia Central Hospital, University of Helsinki, Valto Käkelän Katu 1, 53130 Lappeenranta, Finland

**Keywords:** Biomarkers, Cardiovascular diseases, Acute coronary syndromes

## Abstract

Chronic inflammation plays a crucial role in coronary artery disease (CAD), but differences in specific cytokine profiles between acute coronary syndrome (ACS) and stable CAD remain unknown. We investigated cytokine differences between these two manifestations of CAD. The study included 308 patients with angiographically detected, hemodynamically significant CAD: 150 patients undergone angiography for ACS, 158 patients undergone angiography for stable CAD. To assess dynamic changes, 116 patients had index angiogram at least 3 months earlier. We measured the serum concentrations of 48 circulating cytokines. The ACS group had decreased interleukin (IL) 4 (p = 0.005), and increased IL-8 (p = 0.008), hepatocyte growth factor (HGF) (p < 0.001) and macrophage colony-stimulating factor (M-CSF) (p = 0.002) levels compared with the stable CAD group. Multivariable logistic regression revealed increased levels of HGF (OR 18.050 [95% CI 4.372–74.517], p < 0.001), M-CSF (OR 2.257 [1.375–3.705], p = 0.001) and IL-6 (OR 1.586 [1.131–2.224], p = 0.007), independently associated with ACS. In the post-angiography group, only diminished platelet-derived growth factor-BB levels in ACS-manifested patients were observed (OR 0.478, [0.279–0.818], p = 0.007). Cytokine profiles differ between ACS and stable CAD. Such differences seem to be mainly reversible within 3 months after ACS. Thus, targeting one or two cytokines only might not offer one-size fits all-therapeutic approach for CAD-associated inflammation.

Trial registration: NCT03444259.

## Introduction

Coronary artery disease (CAD) is a major cause of death globally and the incidence is increasing in Western countries^1^. Patients with acute coronary syndromes (ACS) have less favorable long-term prognosis compared to those with stable chronic CAD in terms of sudden cardiac death, myocardial infarction (MI) and repeated revascularization^2,3^. The reasons for this adverse trajectory in patients with ACS remain incompletely understood.

Atherosclerosis is a chronic inflammatory disease^4^. Cytokines are inflammatory modulators that have been shown to affect atherogenesis, plaque rupture, stabilization and atherothrombosis^5^. Therefore, cytokines play a crucial role in the manifestation of CAD. However, it is not known whether cytokines differ in patients with ACS compared to those with stable CAD. Moreover, it is not known whether cytokine profiles stabilize after treatment for ACS to levels observed in patients with stable CAD.

The aim of the present study was to determine the differences in cytokine levels between patients with ACS and those with stable CAD. Our hypothesis was that cytokine profiles differ between these two manifestations of coronary artery disease and could provide novel biomarkers to recognize patients at higher risk of adverse cardiac events.

## Materials and methods

This analysis included patients from two prospective studies, the CAREBANK and the FACT studies, that enrolled patients undergoing diagnostic coronary artery angiography for clinical indications at the Heart Center, Turku University Hospital, Turku, Finland. The present study is registered in ClinicalTrials.gov (ClinicalTrials.gov Identifier: NCT03444259). Both studies were approved by the Ethical Committee of the Hospital District of South-West Finland. Studies conformed with the Declaration of Helsinki as revised in 2002. The patient cohort comprised a total of 469 patients.

**Figure 1:**
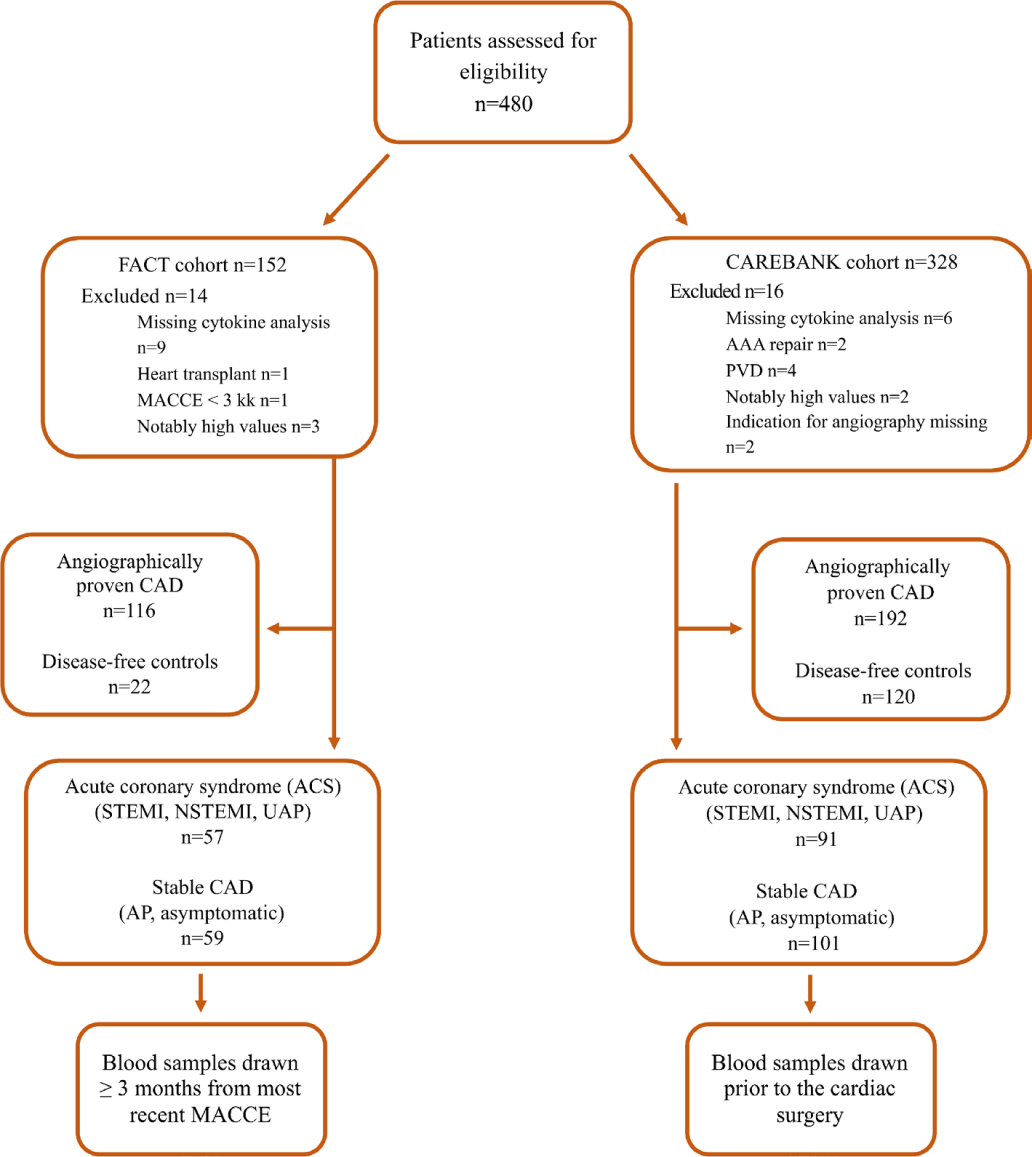
Flow chart of study eligibility and formation of the study cohorts. *AAA* abdominal aortic aneurysm, *AP *angina pectoris, *CAD* coronary artery disease, *MACCE* major adverse cardio and cerebrovascular event, *NSTEMI* non-ST-elevation myocardial infarction, *PVD* peripheral vascular disease, *STEMI* ST-elevation myocardial infarction, *UAP* unstable angina pectoris.

Patient selection from the CAREBANK and the FACT studies for the present analysis is summarized in Fig. 1. CAREBANK is a prospective biobank of patients undergoing adult cardiac surgery (i.e., coronary artery bypass grafting, heart valve surgery, aortic surgery, and resection of cardiac tumors) at the Turku University Hospital since February 2016. Patient records were individually reviewed, and baseline clinical data were collected into a standardized structured datasheet by trained personnel^6^. The study has been monitored by third-party licensed data-monitor. Preoperative study blood samples (ethylenediaminetetraacetic acid treated (EDTA) -plasma and serum) were obtained at the same time with routine sampling by the accredited hospital laboratory. The database is pseudonymized and data privacy regulations have been followed during the study. Written informed consent for the study was obtained from all patients before the study enrolment. Six patients were excluded from the registry due to missing cytokine measurements or failure in ELISA analyses. The CAREBANK study population consisted of 322 patients.

The FACT cohort included consecutive patients who had undergone a diagnostic coronary angiography between September 2015 and December 2015 at the Turku University Hospital. The FACT cohort consisted of patients in whom circulating cytokine levels were measured at least 3 months after the index coronarography. For ACS patients, this enabled the evaluation of cytokines’ dynamic changes after treatment for ACS. Overall, 267 patients fulfilling the inclusion criteria of this study were contacted and 189 of them gave written informed consent. EDTA-plasma and serum samples were taken at certified hospital laboratories. The period between recent major adverse cardiovascular event (MACCE), including nonfatal MI, nonfatal stroke and unscheduled coronary revascularization, and blood sampling was required to be ≥ 3 months. Blood sample was obtained in 152 patients. Altogether, 11 FACT patients were excluded (heart transplant: n = 1; MACCE < 3 months after index coronarography: n = 1; cytokine analyses not available: n = 9). The final FACT cohort consisted of 141 patients.

Five patients had notably high values of various markers and were excluded from the final analyses. We also excluded patients who underwent vascular endoprosthesis operation for abdominal aortic aneurysm (n = 2) or lower limb ischemia (n = 4). Indication for coronary angiography was missing in two patients and they were excluded. Overall, 308 patients had an angiographically proven CAD and were included in this study.

The diagnostic criteria for significant CAD were symptoms related to coronary artery disease and > 50% diameter stenosis in one or more coronary arteries. CAD patients were further divided into two groups: (1) the ACS group, i.e., having ST segment elevation myocardial infarction (STEMI), non-ST segment elevation myocardial infarction (NSTEMI) or unstable angina pectoris (UAP), and the stable CAD group, i.e., having stable angina pectoris. Diabetes, dyslipidemia and hypertension were defined as diseases requiring drug therapy. Based on the smoking history, the patients were classified as never-smoked, ex-smoker, or current smoker.

### Blood sampling and multiplex analysis

Blood sample analyses were conducted at the MediCity Research Laboratory, University of Turku, Turku, Finland. We analyzed the levels of 48 different cytokines by using Bio-Plex panel Pro Human Cytokine Screening Panel, 48-Plex (#12007283, BioRad) according to the manufacturer’s instructions.

### Statistical analysis

Continuous variables were reported as means and standard deviation as well as medians and 25th and 75th interquartile range. Categorical variables are reported as counts and percentages. The Shapiro–Wilk-test was used to examine if the cytokine concentration levels had a normal distribution. As the cytokine levels were not normally distributed, a Mann–Whitney U-test was used to examine the differences between the ACS and the stable CAD groups and between controls. The prevalence of cardiovascular risk factors, medications and other baseline characteristics between the groups were compared using the Chi-square test. Variables with a p < 0.05 in univariate analysis were entered into a logistic regression models, which were used to explore the associations between the clinical variables and cytokine levels. A p-value of < 0.05 was considered statistically significant for clinical variables, and due to nature of the study design, a stricter p-value of < 0.01 was used in cytokine analyzes. Due to small study cohort, no p-value corrections were made to avoid overcorrection. Statistical analyses were performed using the SPSS (IBM SPSS Statistics, SPSS Inc., IBM, Chicago, Illinois, USA) and the R 4.3.1^7^ (for macOS 13.5) statistical softwares.

As the analyzes were conducted in two different study cohorts at different times, we used a Z-score to diminish the potential differences due to batch-induced variability in cytokine analyses between the CAREBANK and FACT cohorts. Z-score was calculated for each cytokine separately according to the following equation:

$$Z-score=\frac{{x}_{i}-\overline{x(controls)}}{SD}$$

where ${x}_{i}$ is cytokine concentration of each patient, $\overline{x(controls)}$ is the mean of cytokine concentrations in healthy controls in each cohort, and SD is standard deviation of cytokine concentration.

## Results

### Study population

The study cohort consisted of 308 patients with angiographically-confirmed CAD. The indication for coronary angiography was an ACS in 150 (48.7%) patients, whereas 158 (51.3%) patients had stable CAD. The proportions of ACS patients were 47.4% and 50.9% in the CAREBANK and FACT cohorts, respectively. Baseline characteristics of these patients are presented in Table 1. Both study cohorts showed a major prevalence of male patients. The prevalence of rheumatic disease was higher in patients with stable disease in CAREBANK cohort, but not amongst FACT patients. On the contrary, stable CAD patients had more frequently heart failure in the FACT cohort, but not in the CAREBANK cohort. Unsurprisingly, the use of aspirin and ADP receptor inhibitors was more common among patients with ACS compared to stable CAD.

**Table 1:** Baseline characteristics of the CAREBANK and the FACT study cohorts.

Baseline characteristics	Carebank			Fact		
	ACS (n = 91)	Stable CAD (n = 101)	P-value	ACS (n = 59)	Stable CAD (n = 57)	P-value
Age (years)	67.65 ± 8.95	67.75 ± 8.79	0.963	69.03 ± 11.77	68.30 ± 9.72	0.821
Female	14 (15.4)	17 (16.8)	0.786	24 (40.7)	15 (26.3)	0.102
Hypertension	81 (89.0)	87 (86.1)	0.548	43 (72.9)	47 (82.5)	0.216
Atrial fibrillation	10 (20.9)	17 (16.8)	0.473	13 (22.0)	16 (28.1)	0.453
Sleep apnea	7 (7.7)	11 (10.9)	0.448	7 (11.9)	12 (21.1)	0.181
Smoking habit						
Current smoker	17 (18.7)	12 (11.9)	0.189	4 (6.9)	6 (10.5)	0.490
Ex-smoker	43 (47.3)	40 (39.6)	0.285	31 (53.4)	26 (45.6)	0.401
Never smoked	31 (34.1)	49 (48.5)	0.043	23 (39.7)	25 (43.9)	0.648
Diabetes						
Type 1 diabetes	6 (6.7)	7 (7.0)	0.928	4 (6.8)	1 (1.8)	0.183
Type 2 diabetes	31 (34.4)	30 (30.0)	0.512	11 (18.6)	18 (31.6)	0.108
Heart failure	15 (16.5)	10 (9.9)	0.176	8 (13.6)	18 (31.6)	0.020
Preoperative creatinine (micromol/L)	112.6 ± 87.7	103.0 ± 68.6	0.920	90.6 ± 32.2	88.6 ± 18.8	0.797
Liver cirrhosis	0 (0.0)	1 (1.0)	0.341	0 (0.0)	0 (0.0)	-
Rheumatic disease	1 (1.1)	11 (10.9)	0.005	10 (16.9)	7 (12.3)	0.477
NYHA classes^a^			0.027			0.003
I	23 (25.3)	28 (27.7)		16 (30.2)	5 (9.1)	
II	32 (35.2)	45 (44.6)		20 (37.7)	26 (47.3)	
III	29 (31.9)	28 (27.7)		11 (20.8)	23 (41.8)	
IV	7 (7.7)	0 (0.0)		6 (11.3)	1 (1.8)	
CCS classes^b^			< 0.001			0.120
I	53 (58.2)	47 (46.5)		25 (46.3)	28 (50.9)	
II	13 (14.3)	39 (38.6)		16 (29.6)	13 (23.6)	
III	21 (23.1)	15 (14.9)		7 (13.0)	13 (23.6)	
IV	4 (4.4)	0 (0.0)		6 (11.1)	1 (1.8)	
Medications						
Treatment for dyslipidemia	84 (92.3)	90 (89.1)	0.448	57 (96.6)	54 (94.7)	0.619
Treatment for diabetes	38 (41.8)	38 (37.6)	0.559	15 (25.4)	19 (33.3)	0.349
Insulin therapy	20 (22.0)	20 (19.8)	0.711	7 (11.9)	6 (10.5)	0.819
Antithrombotic drugs						
Warfarin	10 (11.0)	9 (8.9)	0.630	8 (13.6)	14 (24.6)	0.131
DOAC	6 (6.6)	3 (3.0)	0.236	3 (5.1)	4 (7.0)	0.662
ASA	74 (81.3)	70 (69.3)	0.055	52 (88.1)	36 (63.2)	0.002
ADP receptor inhibitor	16 (17.6)	1 (1.0)	< 0.001	47 (79.7)	29 (50.9)	0.001
Calcium channel blocker	25 (27.5)	40 (39.6)	0.076	9 (15.3)	13 (22.8)	0.300
Beta-blockers	73 (80.2)	71 (70.3)	0.113	43 (72.9)	46 (80.7)	0.319
ACEis/ARBs	66 (72.5)	67 (66.3)	0.353	42 (71.2)	46 (80.7)	0.231

Continuous variables are reported as mean ± standard deviation. Categorical variables are reported as counts and percentages (in parentheses).

*ACEi* angiotensin-converting enzyme inhibitor, *ACS* acute coronary syndrome, *ADP* adenosine diphosphate, *ARB *angiotensin receptor blocker, *ASA *acetylsalicylic acid, *CAD *coronary artery disease, *CCS *Canadian Cardiovascular Society, *DOAC *direct oral anticoagulant, *NYHA *New York Heart Association.

^a^Data missing from 8 patients of the FACT study.

^b^Data missing from 7 patients of the FACT study.

### Predictors for acute coronary syndrome

The levels of interleukin 2 (IL-2), IL-15, interleukin-12 subunit p40 (IL-12p40) and monocyte-chemotactic protein 3 (MCP-3) were mostly below the detection limit of the assay.

**Figure 2:**
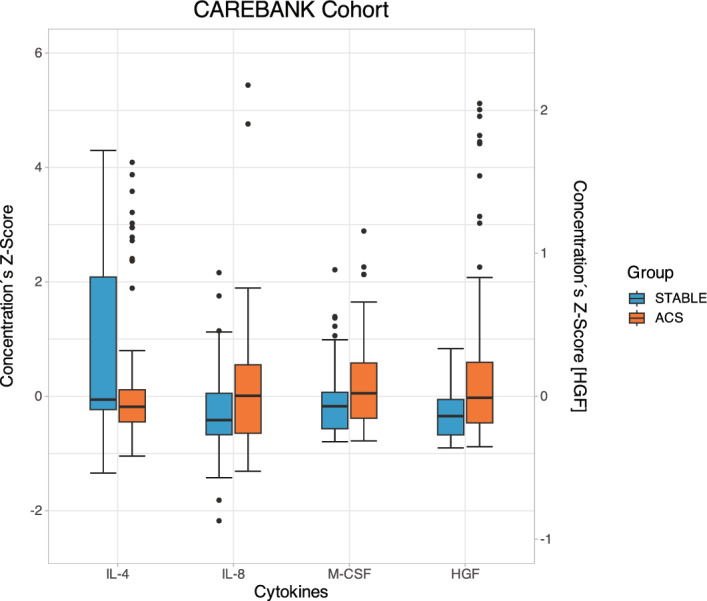
Cytokine levels of IL-4, IL-8, M-CSF and HGF in the CAREBANK study cohort. Data are shown as medians and 25th and 75th percentiles. The vertical axis represents cytokine concentration’s Z-scores and horizontal axis different cytokines in the acute coronary syndrome (ACS) group (orange) and stable coronary artery disease (STABLE) group (blue). Thirteen outliers were excluded from the figure. *ACS* acute coronary syndrome, *IL* interleukin, *M-CSF* macrophage colony-stimulating factor, *HGF *hepatocyte growth factor.

Figure 2 summarizes the levels of significant circulating cytokine levels in the CAREBANK cohort. Compared to patients with stable CAD, patients with ACS had decreased levels of IL-4 (p = 0.005) and increased levels of hepatocyte growth factor (HGF) (p < 0.001), macrophage colony-stimulating factor (M-CSF) (p = 0.002) and IL-8 (p = 0.008). The levels of interleukin-2 receptor subunit alpha (IL-2Rα) (p = 0.013), IL-6 (p = 0.018), macrophage inflammatory protein 1 alpha (MIP-1α) (p = 0.018), stem cell growth factor beta (SCGF-β) (p = 0.033) and tumor necrosis factor beta TNF-β (p = 0.035) were generally higher and level of eotaxin (p = 0.025) lower in ACS patients (p < 0.050). The results of statistical analyses are summarized in Table 2.

**Table 2:** The Z-scores of cytokines that achieved a p-value < 0.05 in univariate analysis in the CAREBANK cohort or in the FACT cohort.

Cytokines	CAREBANK			FACT			
	ACS Median (IQR)	Stable CAD Median (IQR)	P-value	ACS Median (IQR)	Stable CAD Median (IQR)		P-value
HGF	− 0.010 (− 0.186 to 0.238)	− 0.139 (− 0.270 to − 0.022)	< 0.001	− 0.056 (− 0.443 to 0.817)	0.175 (− 0.513 to 1.045)		0.581
M− CSF	0.060 (− 0.380 to 0.610)	− 0.175 (− 0.566 to 0.070)	0.002	− 0.047 (− 0.448 to 0.629)	− 0.047 (− 0.391 to 0.565)		0.836
IL− 4	− 0.182 (− 0.449 to 0.115)	− 0.057 (− 0.233 to 2.086)	0.005	0.052 (− 0.395 to 0.678)	0.014 (− 0.328 to 0.678)		0.938
IL-8	0.011 (− 0.645 to 0.552)	− 0.417 (− 0.671 to 0.053)	0.008	− 0.16 (− 0.210 to − 0.101)	− 0.149 (− 0.204 to − 0.068)		0.577
IL-2Rα	0.165 (− 0.419 to 0.583)	− 0.148 (− 0.697 to 0.270)	0.013	0.001 (− 0.355 to 0.409)	− 0.192 (− 0.417 to 0.493)		0.398
IL-6	0.053 (− 0.495 to 0.667)	− 0.148 (− 0.559 to 0.291)	0.018	− 0.270 (− 0.412 to − 0.058)	− 0.201 (− 0.375 to 0.602)		0.127
MIP-1α	− 0.082 (− 0.159 to 0.000)	− 0.150 (− 0.218 to − 0.042)	0.018	− 0.342 (− 0.620 to 0.341)	− 0.08 (− 0.516 to 0.385)		0.203
Eotaxin	− 0.225 (− 0.694 to 0.464)	0.030 (− 0.372 to 0.901)	0.025	0.222 (− 0.591 to 1.467)	0.089 (− 0.563 to 0.564)		0.164
SCFG-β	− 0.145 (− 0.779 to 0.255)	− 0.467 (− 0.816 to 0.015)	0.033	0.328 (− 0.604 to 1.305)	0.701 (− 0.275 to 2.169)		0.125
TNF-β	− 0.164 (− 0.620 to 0.596)	− 0.377 (− 0.857 to 0.167)	0.035	− 0.042 (− 0.487 to 0.452)	− 0.042 (− 0.512 to 0.484)		0.860
PDGF-BB	0.050 (− 0.606 to 0.570)	0.172 (− 0.389 to 0.540)	0.422	− 0.079 (− 0.497 to 0.417)	0.24 (− 0.305 to 0.925)		0.024

Data are shown as medians and 25th–75th interquartile range (IQR).

*ACS* acute coronary syndrome, *CAD* coronary artery disease, *HGF* hepatocyte growth factor, *IL-2Rα* interleukin-2 receptor subunit alpha, *IQR* 25th–75th interquartile range, *M-CSF* macrophage colony-stimulating factor, *MIP-1α* macrophage inflammatory protein 1 alpha, *PDGF-BB* platelet-derived growth factor-BB, *SCGF-β* stem cell growth factor beta, *TNF-β* tumor necrosis factor beta.

Multivariable logistic regressions adjusted for rheumatic disease and history of never smoking in the CAREBANK patients showed increased levels of HGF (OR 18.050, 95% CI 4.372–74.517, p < 0.001), M-CSF (OR 2.257, 95% CI 1.375–3.705, p = 0.001) and IL-6 (OR 1.586, 95% CI 1.131–2.224, p = 0.007) in ACS patients (p < 0.01). Using a significance level of p < 0.05, also IL-4 (OR 0.739, 95% CI 0.587–0.930, p = 0.010), interferon gamma (IFN-γ) (OR 0.748, 95% CI 0.593–0.944, p = 0.015), IL-8 (OR 1.591, 95% CI 1.081–2.341, p = 0.019), IL-17 (OR 0.699, 95% CI 0.516–0.945, p = 0.020), granulocyte–macrophage colony-stimulating factor (GM-CSF) (OR 0.741, 95% CI 0.560–0.981, p = 0.036), fibroblast growth factor-basic (FGFbasic) (OR 0.595, 95% CI 0.363–0.976, p = 0.040), IL-3 (OR 1.609, 95% CI 1.009–2.564, p = 0.046) and β-NGF (OR 1.434, 95% CI 1.003–2.050, p = 0.048) were independent predictors of ACS.

### Comparison 3 months after the index angiogram

ACS patients in the FACT cohort represent those with most recent MACCE at least 3 months earlier. No differences were seen in the levels of cytokines between ACS and stable CAD patients. However, a logistic regression model adjusted for heart failure showed an association between ACS and diminished levels of platelet-derived growth factor B’s (PDGF-B) active form of homozygous dimer PDGF-BB (OR 0.478, 95% CI 0.279–0.818, p = 0.007), increased levels of eotaxin (OR 1.538, 95% CI, 1.089–2.172, p = 0.015) and stem cell factor (SCF) (OR 2.258, 95% CI 1.078–4.728, p = 0.031).

### ACS and stable CAD patients compared to disease-free controls

There were 120 patients in CAREBANK study cohort and 22 patients in the FACT cohort with no significant CAD detectable in invasive coronary angiography. We used these patients as disease-free controls to examine how cytokine profiles differ when these two different manifestations of CAD are compared to disease-free controls. We analyzed both study cohorts separately to reduce the potential differences due to batch-induced variability in cytokine analyses. Baseline characteristics of disease-free controls are presented in Supplementary Table S1.

In the CAREBANK cohort, ACS patients had significantly higher levels of HGF (p = 0.002) compared to disease-free controls while M-CSF (p = 0.010), IL-6 (p = 0.011), TNF-α (p = 0.013) and MIP-1β (p = 0.029) achieved a p-value < 0.050 with higher levels in ACS patients. Stable patients had elevated levels of IL-4 (p < 0.001) and IL-17 (p = 0.004) compared to disease-free controls, while IFN-γ (p = 0.014), IL-10 (p = 0.022), IL-1β (p = 0.023), PDGF-BB (p = 0.035) and eotaxin (p = 0.040) were generally higher and level of stem cell growth factor-beta (SCGF-β) (p = 0.019) lower in patients with stable disease. Cytokine levels are presented in Supplementary Fig. S1 and S2.

In the FACT cohort, both ACS and stable CAD patients had higher levels of IL-18 compared to controls (p = 0.002 and 0.011, respectively). Stable CAD patients also had higher concentration of IL-9 (p = 0.005). In contrast to the CAREBANK cohort, the level of SCGF-β was elevated in stable disease compared to controls (p = 0.025). Cytokine levels are presented in Supplementary Fig. S3. Cytokine statistics are presented in more detail in Supplementary Tables S2 and S3.

In multivariable logistic regressions adjusted for age, sex, hypertension, treatment for dyslipidemia and diabetes, and in stable CAD patients also with atrial fibrillation, elevated levels of IL-4 (p = 0.003), IFN-γ (0.009) and lower level of MCP-3 (p = 0.009) and SCGF-β (p = 0.006) were independent predictors for stable CAD compared to controls in the CAREBANK cohort. In ACS patients, TNF-α reached a p-value of 0.011 and higher concentrations predicted ACS. In the FACT cohort, multivariable logistic regressions adjusted for atrial fibrillation, history of smoking, rheumatic disease and treatment for dyslipidemia, elevated levels of IL-17 (p = 0.007) and IL-18 (p = 0.003) were predictors of ACS. In a regression model adjusted with treatment for dyslipidemia, elevated level of IL-18 was also associated with stable disease. All cytokines that achieved a p-value < 0.05 are presented in Supplementary Table S4.

### Atherosclerotic burden and cytokine levels

Atherosclerotic burden is a possible factor that could affect cytokine levels. The extent of atherosclerosis was not systematically assessed among the CAREBANK cohort i.e. using a SYNTAX Score. Therefore, we analyzed the extent of coronary artery disease using number of distal anastomoses as surrogate for more advanced disease in CAREBANK patients.

We divided the patients into two groups according to the number of distal anastomoses: patients with “minor” disease burden (one or two anastomoses) and “severe” disease burden (three or more). Information was available on 187 patients, of which 54 had one or two anastomoses and 133 patients had three or more anastomoses. Baseline characteristics were similar between the groups, as only use of aspirin and statin were more common in severe group (data not shown).

Amongst ACS patients, 26 patients had minor (28.6%) and 65 patients severe disease burden, and amongst stable CAD patients, 27 (29.7%) had minor and 65 severe disease burden. There was no difference in disease severity between ACS and stable groups (p = 0.908). There was no significant difference in cytokine levels between these disease burden categories in total cohort, nor in ACS or stable groups separately (data not shown).

## Discussion

The study showed that patients with ACS have distinct circulating cytokine profiles compared to patients with stable CAD and that these differences diminish during the course of few months after the cardiac event. This may indicate that the majority of cytokines return to similar levels with stable CAD and new cytokines emerge within 3 months. This suggests that these cytokines have various roles in the pathobiology of an acute coronary event and plaque stability.

ACS usually results from atherosclerotic plaque rupture or plaque erosion and the following coronary artery thrombosis that obstructs blood flow leading to myocardial infarction^8^. Macrophages have an influence on all atherosclerosis-related processes from initiation to progression, and plaque vulnerability^9^. Macrophages can be divided into classical M1-phenotype and alternative M2-macrophages^9^. Pro-inflammatory cytokines, such as IFN-γ and IL-1β, favor M1-polarization and M1-macrophages produce inflammatory cytokines, e.g. IL-6, IL-12 and TNF-α^10^. M1 macrophages contribute to an increased and sustained inflammatory response and enduring M1 macrophage activation can cause tissue damage and impair wound healing^9^. M2-macrophages are considered anti-inflammatory and are induced by anti-inflammatory cytokines, such as IL-4, IL-13 and IL-10^11^. M2-polarized macrophages promote tissue repair and healing, angiogenesis, debris scavenging and production of anti-inflammatory cytokines e.g. IL-1 receptor antagonist (IL-1ra), IL-4, IL-10, IL-13 and TGF-β^11,12^. Histologically there is no significant difference between M1- and M2- polarized macrophages in the fibrous cap of an atherosclerotic lesion, but M1-macrophages are overrepresented in the rupture-prone shoulder lesions of the plaque^13^. This supports the comprehension of inflammation’s negative impact on plaque stability.

Many cytokines affect platelet aggregation and thrombus formation, and therefore create an important connection between inflammation and coagulation^14,15^. Plaque rupture exposes blood and its coagulation factors to the thrombogenic material of the plaque interior, thus leading to thrombus formation^16^. Proinflammatory cytokines, like IL-1, IL-6 and TNF-α, activate the expression of tissue factor on blood cells and dampen the natural anticoagulant pathways, e.g. the protein C pathway, that normally limits the anticoagulation response, thereby favoring thrombus formation^17,18^. Activated platelets release inflammatory mediators, such as IL-1β, IL-8 and other chemokines, that in turn recruit monocytes and neutrophils to lesion site, thus enhancing the inflammation in the ruptured area^19,20^.

Cardiovascular disease remains as a major cause of death worldwide. The amount of ST-elevation myocardial infarction (STEMI) patients without any standard modifiable cardiovascular risk factors (SMuRFs: hypertension, diabetes mellitus, hypercholesterolemia, smoking) is increasing^21^. Hence, there is a clinical need to recognize these SMuRFless patient with elevated risk of ACS. Cytokines could act as biomarkers to identify these patients.

IL-6 is a widely studied cytokine with both pro-inflammatory and anti-inflammatory properties^22^. IL-6 has a complex role in cardiovascular system but its cumulative action results in progression of ischemic heart disease^23^. Similar to our study, elevated levels of circulating IL-6 have been reported in ACS patients^24^, and elevated levels also predict adverse events^25^. In our study, the level of IL-4 was decreased in ACS patients compared to those with stable disease and the decreased level was also associated with ACS in logistic regression model. IL-4 is generally considered as an anti-inflammatory cytokine, but there is also increasing evidence that IL-4 has pro-inflammatory effects on vascular endothelium^26^. There is limited number of studies considering circulating IL-4 levels in ACS patients. Luo et al.^27^ reported elevated IL-4 in STEMI patients compared to stable angina pectoris patients and Zhao et al.^28^ reported similar results when ACS patients were compared to healthy individuals. On the other hand, elevated level of IL-4 has also been associated with reduced risk of CVD^29^.

IL-8 is a proinflammatory cytokine with pro-atherogenic properties and a suspected role in plaque destabilization^30^. In concordance to our findings, previous studies have reported ACS patients to have higher levels of circulating IL-8 compared to stable CAD patients and healthy controls^31,32^. Elevated levels have been associated with greater infarct size, lower left ventricle (LV) ejection fraction and higher probability of adverse events in STEMI patients^33^, higher all-cause mortality in ACS patients during a 5-year follow up^34^ and increased risk of future CAD among apparently healthy subjects^30^. Due to its role in ACS and its complications, IL-8 could be a potent target for future pharmacological developments.

In addition to inflammation, also angiogenesis, neovascularization and endothelial cell apoptosis are known to have an important role in atherosclerotic plaque formation, disease progression and plaque stability^8^. Hepatocyte growth factor (HGF) is a pro-angiogenic growth factor, that regulates endothelial cell growth, inhibits apoptosis, promotes angiogenesis, and is expressed in atherosclerotic plaques^35^. Animal models have shown that HGF has cardioprotective properties^36,37^, and elevated levels have been reported in acute MI patients^38^. This has led to presumption that the increase in HGF level is a defensive response to myocardial injury^39^. On the other hand, elevated circulating HGF has also been associated with greater progression of atherosclerosis in 12-year follow-up^40^, though it might only be a biomarker of proceeding atherosclerosis, not the cause of it. Santalahti et al. discovered that high plasma levels of HGF predicted all-cause and CVD mortality in 10-year follow-up^41^. In our study, HGF was elevated in ACS patients in early phase but not after few months of MACCE. This is likely due to fairly short half-time, as Sato el al. have previously reported that the level of HGF decline to less than half in just over one day^42^.

Macrophages and monocytes are important in the pathogenesis of atherosclerosis as phagocytosis of lipids by macrophages and the following formation of foam cells initiate development of atherosclerotic lesions^43^. Macrophage colony-stimulating factor (M-CSF) is a growth factor that promotes the development of monocytes and macrophages from their hematopoietic pre-cursor cells^44^. M-CSF has an influence on atherogenesis by increasing monocyte number and macrophage proliferation, survival and differentiation into M2-polarized phenotype, monocyte migration to inflammatory sites, and lipid uptake by macrophages^44^. M-CSF levels have been detected to be higher in atherosclerotic arteries compared to normal arteries^45^. Alike to our findings, Saitoh et al. documented elevated levels of M-CSF in UAP patients compared to stable AP and also showed a positive correlation between M-CSF level and future coronary events^46^.

There is a distinct difference in cytokine profiles between our two study cohorts and therefore at different time points of acute coronary syndrome. PDGF-BB was the only cytokine that was associated with ACS in the FACT cohort (MACCE over 3 months) when the level of significance was p < 0.010, with a lower concentration predicting ACS. This is in contrast to a previous study of Tahara et al., who discovered that PDGF-B was significantly elevated in AMI patients in the chronic phase (after 1–4 months after AMI) even though the levels were lower in acute phase compared to stable AP patients and controls^47^. Like M-CSF, PDGF-BB is also a growth factor, and it influences smooth muscle cell proliferation and migrations as well as vasoconstriction^48^. Animal studies with AMI models have shown that administration of exogenous PDGF-BB decreases infarct size and enhance LV ejection fraction^47,49^. Autopsy study of ACS patients showed that PDGF-B was highly expressed in macrophages and smooth muscle cells (SMC) at lesion sites on days 24–55 after PCI but decreased after 6 months, suggesting PDGF-BB might have a role in intima repair^50^.

Our study has certain limitations that we are aware of. First, the study population is small compared to the number of cytokines measured. Therefore, we decided to use a more stringent p-value < 0.010 as a level of significance. Blood samples were obtained preoperatively, not at admission to the hospital. Also, some cytokines have a short elimination time. Therefore, the most acute phase of ACS might have passed involving some ACS patients. In the FACT cohort, the disease-free control group only consisted of 22 patients, and this must be regarded when interpreting results. Especially in the CAREBANK cohort, many disease-free controls had a valvular defect as an indication for open-heart operation, which may affect the cytokine levels. Preoperative creatinine levels differed between the CAREBANK and FACT cohorts. Within these two study cohorts separately, there was no difference in creatinine levels between ACS and stable patients. It is unlikely that slightly higher creatinine levels would explain the presence of differences in cytokine profiles between ACS and stable patients only in the CAREBANK cohort. Malignancy and obesity were not registered from the whole study population, and thus, these variables were not taken into account in the analyses.

We also acknowledge that this is not a pure longitudinal study as blood samples after three months since most recent MACCE were obtained from different cohorts. However, the baseline characteristics and the proportions of ACS and stable patients were similar between these study cohorts. Thus, the most straight-forward interpretation is that the differences seen in cytokine profiles between the CAREBANK and FACT cohorts represent average cytokine trends after the most recent MACCE.

We used the number of distal anastomoses as a surrogate for disease extent as other measurements of atherosclerotic burden were not systematically collected. We understand that this is uncommon, and e.g. SYNTAX or Gensini score would be more accurate. However, cytokine profiles did not differ between minor and severe disease burden. This reinforces our findings, that differences between ACS and stable patients are due to acute coronary event rather than extent of the disease.

To summarize, the present study suggests that certain cytokines might have a pivotal role in the development of ACS and MI. Our results may imply that targeting single or only few cytokines does not offer a general therapeutic approach for the whole spectrum of atherosclerosis-related inflammation resolution. It is possible that the need and target of immunomodulation changes along with the clinical course (ACS vs. stable CAD) and other aspects of the disease. Identifying the cytokines involved in the pathophysiology of ACS could provide a novel pharmacological approach for treatment of CAD and prevention of ACS. Additional research is required to determine if cytokines could provide novel biomarkers for patients at risk of an acute coronary event.

### Supplementary Information

Supplementary Information.

## Data Availability

The datasets generated during and/or analysed during the current study are available from the corresponding author on reasonable request.
