# Supplementary material for: Differential circulating cytokine profiles in acute coronary syndrome versus stable coronary artery disease
Source: Sci Rep. 2024 Jul 27;14:17269. doi: 10.1038/s41598-024-68333-7 (PMC11283453; doi:10.1038/s41598-024-68333-7)
Supplement: Supplementary file 1 — Supplementary Information. [file 41598_2024_68333_MOESM1_ESM.pdf]

Supplementary Information

Differential Circulating Cytokine Profiles in Acute Coronary Syndrome Versus Stable Coronary Artery Disease

Eveliina Maaniitty, Juho Jalkanen, Sami Sinisilta, Jarmo Gunn, Tuija Vasankari, Fausto Biancari, Sirpa Jalkanen, K.E. Juhani Airaksinen, Maija Hollmén, Tuomas Kiviniemi

Supplementary Figure S1. Cytokine levels of MIP-1β, TNF-α, IL-17, IFN-γ, eotaxin and HGF in the CAREBANK study cohort.

Supplementary Figure S2. Cytokine levels of IL-1β, IL-4, IL-6, IL-10, M-CSF and SCGF-β in the CAREBANK study cohort.

Supplementary Figure S3. Cytokine levels of IL-9, IL-18 and SCGF-β in the FACT study cohort.

Supplementary Table S1. Baseline characteristics of disease-free control patients in both CAREBANK and FACT study cohorts.

Supplementary Table S2. Cytokine concentration's Z-scores of cytokines that achieved a p-value <0.05 in the CAREBANK cohort.

Supplementary Table S3. Cytokine concentration's Z-scores of cytokines with p-value <0.05 in the FACT cohort.

Supplementary Table S4. P-values and risk estimates from adjusted logistic regression models in both study cohorts compared to disease-free controls.

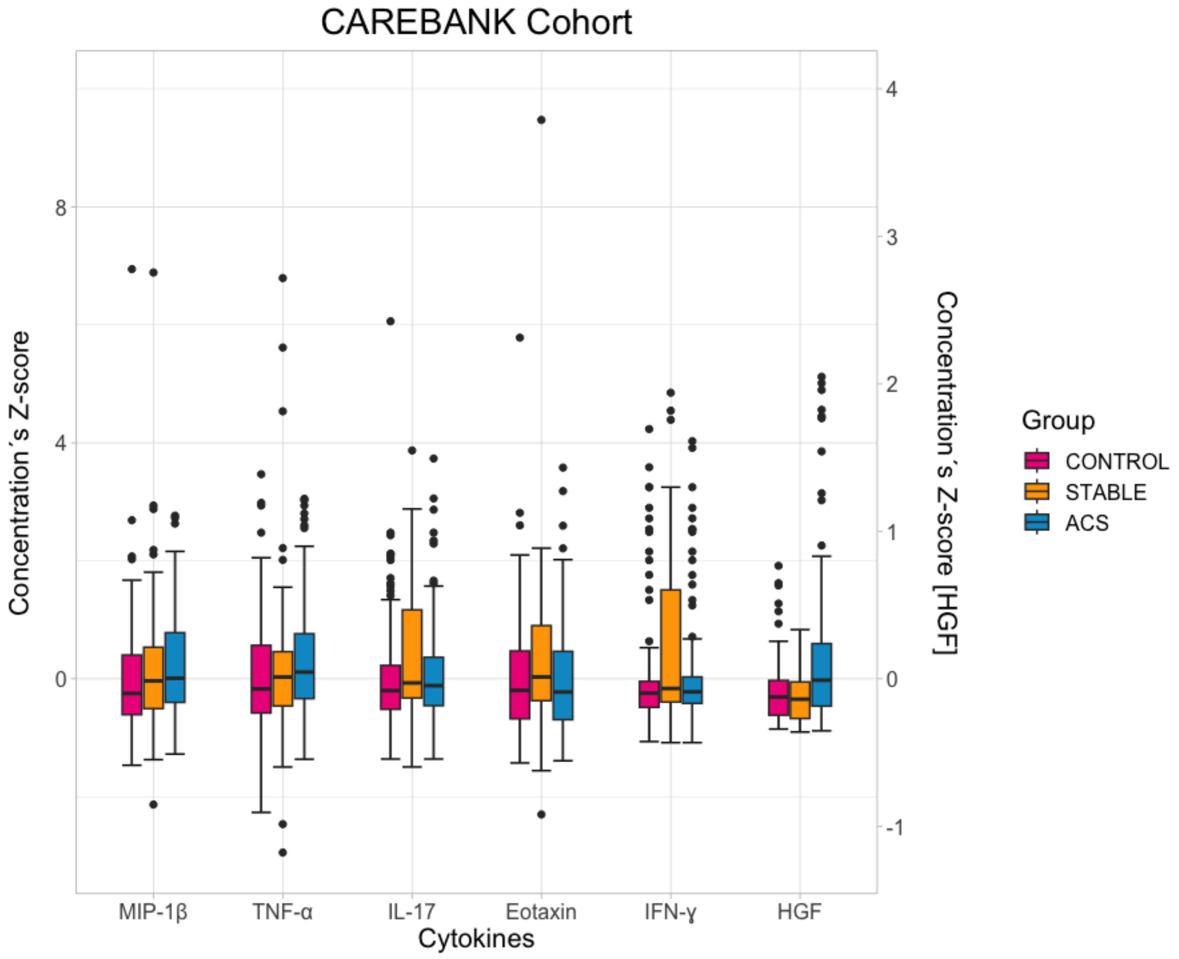

Supplementary Figure S1. Cytokine levels of MIP-1β, TNF-α, IL-17, IFN-γ, eotaxin and HGF in the CAREBANK study cohort. Data are shown as medians and 25th and 75th percentiles. The vertical axis represents cytokine concentration's Z-scores and horizontal axis different cytokines in the acute coronary syndrome (ACS) group (blue), stable coronary artery disease group (orange) and disease-free controls (magenta). Ten outliers were excluded from the figure. PDGF-BB is not shown due to different magnitude of cytokine concentrations.

Abbreviations: ACS= acute coronary syndrome; IFN-γ= interferon gamma; IL=interleukin; HGF= hepatocyte growth factor; MIP-1β= macrophage inflammatory protein 1 beta; TNF-α= tumor necrosis factor alpha.

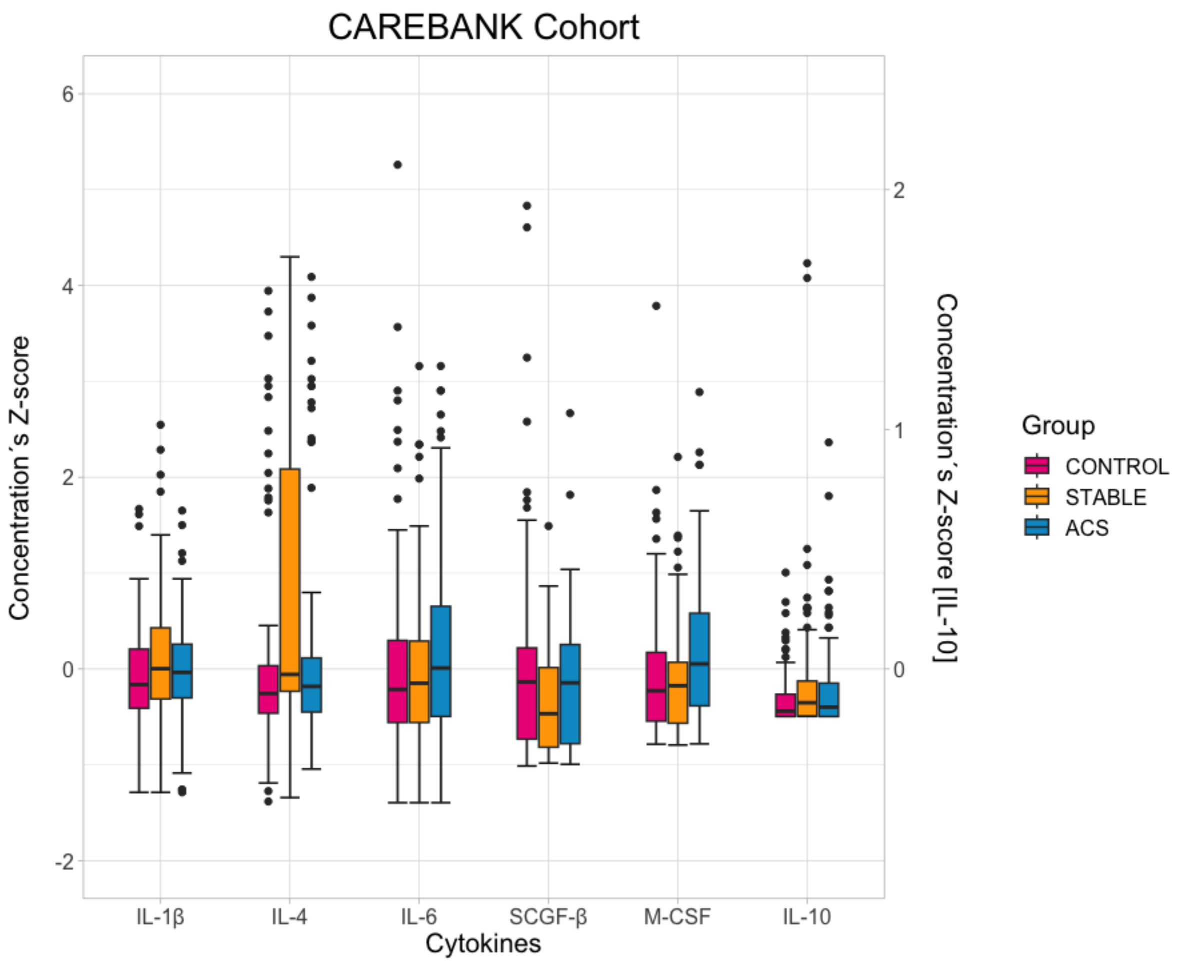

Supplementary Figure S2. Cytokine levels of IL-1β, IL-4, IL-6, IL-10, M-CSF and SCGF-β in the CAREBANK study cohort.

Data are shown as medians and 25 th and 75 th percentiles. The vertical axis represents cytokine concentration's Z-scores and horizontal axis different cytokines in the acute coronary syndrome (ACS) group (blue), stable coronary artery disease group (STABLE) (orange) and disease-free controls (magenta). 25 outliers were excluded from the figure.

Abbreviations: ACS= acute coronary syndrome; IL=interleukin; M-CSF= macrophage colony-stimulating factor; SCGF-β= stem cell growth factor beta.

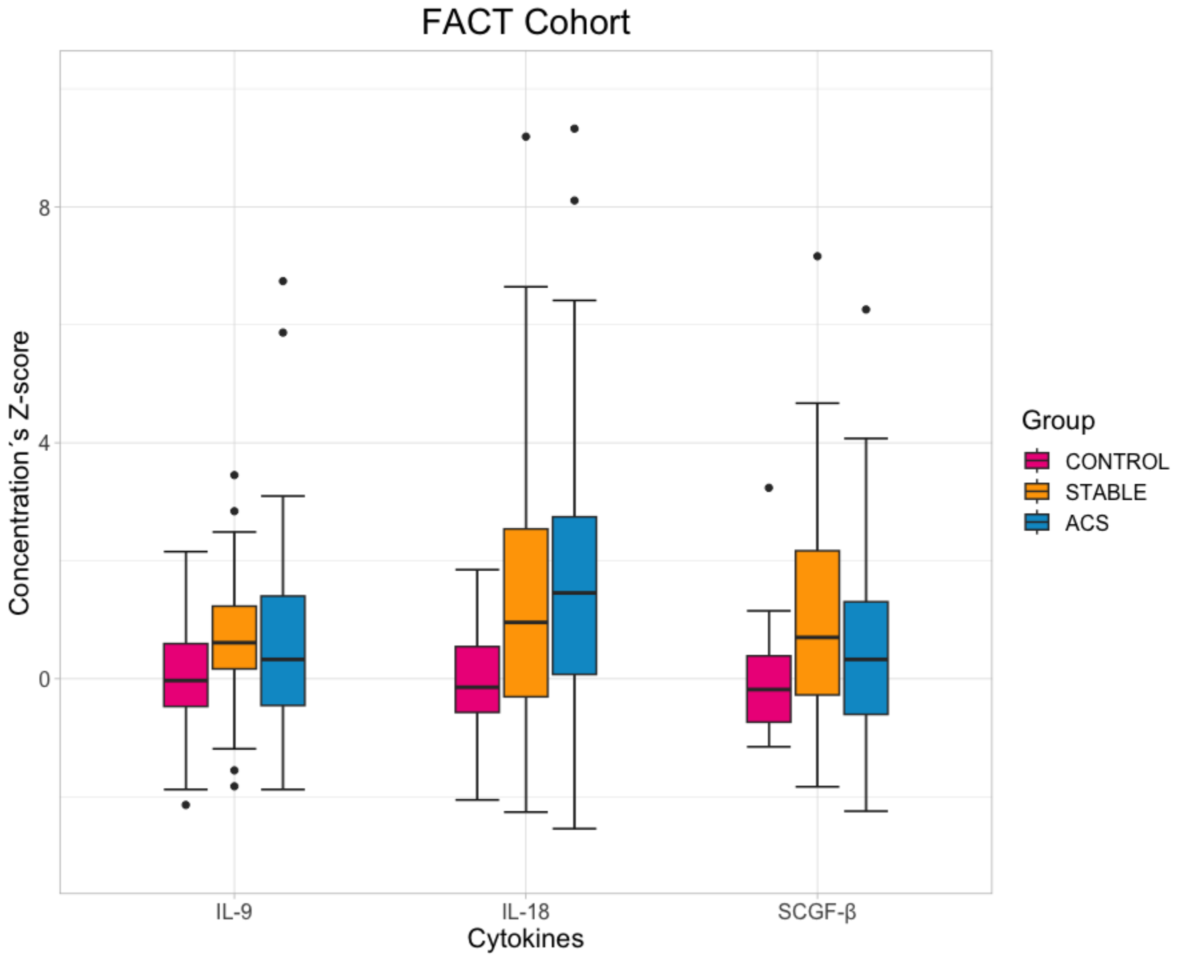

Supplementary Figure S3. Cytokine levels of IL-9, IL-18 and SCGF-β in the FACT study cohort. Data are shown as medians and 25th and 75th percentiles. The vertical axis represents cytokine concentration's Z-score and horizontal axis different cytokines in the acute coronary syndrome (ACS) group (blue), stable coronary artery disease group (STABLE) (orange) and disease-free controls (magenta). Eight outliers were excluded from the figure.

Abbreviations: ACS= acute coronary syndrome; IL=interleukin; SCGF-β= stem cell growth factor beta.

Supplementary Table S1. **Baseline characteristics of disease-free control patients in both CAREBANK and FACT study cohorts.**

Baseline characteristics	CAREBANK			FACT		
	CONTROLS (n=120)	P-value ACS	P-value Stable CAD	CONTROLS (n=22)	P-value ACS	P-value Stable CAD
Age (years)	62.39 ± 12.99	0.011	0.010	66.10 ± 10.14	0.199	0.163
Female	36 (30.0)	0.013	0.022	9 (40.9)	0.985	0.206
Hypertension	77 (64.2)	<0.001	<0.001	16 (72.7)	0.989	0.335
Atrial fibrillation	34 (28.3)	0.216	0.043	11 (50.0)	0.014	0.065
Sleep apnea	8 (6.7)	0.774	0.264	1 (4.5)	0.326	0.076
Smoking habit						
Current smoker	22 (18.3)	0.949	0.185	3 (13.6)	0.341	0.697
Ex-smoker	44 (36.7)	0.122	0.654	6 (27.3)	0.036	0.137
Never smoked	54 (45.0)	0.109	0.602	13 (59.1)	0.119	0.225
Diabetes						
Type 1 diabetes	0 (0.0)	0.004	0.003	0 (0.0)	0.210	0.532
Type 2 diabetes	17 (14.2)	<0.001	0.004	4 (18.2)	0.962	0.234
Heart failure	21 (17.5)	0.846	0.105	7 (31.8)	0.060	0.984
Preoperative creatinine (micromol/L)	93.31 ± 21.75	0.347	0.482	86.24 ± 22.22	0.523	0.386
Liver cirrhosis	0 (0.0)	-	0.275	0 (0.0)	-	-
Rheumatic disease	7 (5.8)	0.075	0.171	0 (0.0)	0.039	0.085
NYHA classes[a]		0.031	0.187		0.773	0.019
I	46 (38.3)			8 (38.1)		
II	45 (37.5)			7 (33.3)		
III	27 (22.5)			5 (23.8)		
IV	2 (1.7)			1 (4.8)		
CCS classes[b]		<0.001	<0.001		0.006	0.007
I	114 (95.0)			19 (90.5)		
II	3 (2.5)			1 (4.8)		
III	3 (2.5)			0 (0.0)		
IV	0 (0.0)			1 (4.8)		
**Medications**						
Treatment for dyslipidemia	35 (29.2)	<0.001	<0.001	9 (40.9)	<0.001	<0.001
Treatment for diabetes	17 (14.2)	<0.001	<0.001	4 (18.2)	0.494	0.184
Insulin therapy	3 (2.5)	<0.001	<0.001	1 (4.5)	0.326	0.402
Antithrombotic drugs						
Warfarin	22 (18.3)	0.141	0.045	8 (36.4)	0.022	0.294
DOAC	10 (8.3)	0.636	0.091	2 (9.1)	0.505	0.755
ASA	22 (18.3)	<0.001	<0.001	6 (27.3)	<0.001	0.004
ADP receptor inhibitor	1 (0.8)	<0.001	0.902	1 (4.5)	<0.001	<0.001
Calcium channel blocker	25 (20.8)	0.261	0.002	3 (13.6)	0.855	0.363
Beta-blockers	67 (55.8)	<0.001	0.027	14 (63.6)	0.418	0.112
ACEis/ARBs	63 (52.5)	0.003	0.037	16 (72.7)	0.891	0.439

Continuous variables are reported as mean ± standard deviation. Categorical variables are reported as counts and percentages (in parentheses). P-values are from Chi-square test between ACS patients and control patients (*P-value ACS*) and between Stable CAD patients and control patients (*P-value Stable CAD*).

Abbreviations: ACEi = angiotensin-converting enzyme inhibitor; ACS= acute coronary syndrome; ADP= adenosine diphosphate; ARB = angiotensin receptor blocker; ASA = acetylsalicylic acid; CAD = coronary artery disease; CCS = Canadian Cardiovascular Society; DOAC = direct oral anticoagulant; NYHA = New York Heart Association.

[a]: data missing from 1 patients of the FACT study.

[b]: data missing from 1 patients of the FACT study.

Supplementary Table S2. Cytokine concentration's Z-scores of cytokines that achieved a p-value <0.05 in the CAREBANK cohort.

Cytokines		CAREBANK			
	ACS Median (IQR)	Stable CAD Median (IQR)	Controls Median (IQR)	P-value ACS	P-value STABLE
IL-4	-0.182 (-0.449–0.115)	-0.057 (-0.233–2.086)	-0.256 (-0.462–0.034)	0.350	<0.001
HGF	-0.010 (-0.186–0.238)	-0.139 (-0.270– -0.022)	-0.122 (-0.247– -0.009)	0.002	0.411
IL-17	-0.117 (-0.457–0.364)	-0.071 (-0.327–1.168)	-0.201 (-0.518–0.226)	0.242	0.004
M-CSF	0.060 (-0.380–0.61)	-0.175 (-0.566–0.070)	-0.217 (-0.544–0.181)	0.010	0.496
IL-6	0.053 (-0.495–0.667)	-0.148 (-0.559–0.291)	-0.214 (-0.559–0.298)	0.011	0.839
TNF-α	0.113 (-0.337–0.763)	0.113 (-0.462–0.459)	-0.172 (-0.581–0.567)	0.013	0.146
IFN-γ	-0.222 (-0.418–0.031)	-0.166 (-0.395–1.506)	-0.245 (-0.485– -0.045)	0.396	0.014
SCGF-β	-0.145 (-0.779–0.255)	-0.467 (-0.816–0.015)	-0.136 (-0.732–0.220)	0.959	0.019
IL-10	-0.159 (-0.198– -0.059)	-0.141 (-0.196– -0.050)	-0.176 (-0.198– -0.098)	0.534	0.022
IL-1β	-0.036 (-0.302–0.259)	0.003 (-0.303–0.451)	-0.162 (-0.409–0.208)	0.107	0.023
MIP-1β	0.008 (-0.401–0.781)	-0.036 (-0.506–0.534)	-0.249 (-0.61–0.403)	0.029	0.293
PDGF-BB	0.050 (-0.606–0.57)	0.172 (-0.389–0.540)	-0.200 (-0.631–0.388)	0.175	0.035
Eotaxin	-0.225 (-0.694–0.464)	0.030 (-0.372–0.901)	-0.196 (-0.679–0.471)	0.837	0.040

Data are shown as medians and 25th-75th interquartile range (IQR). P-values are from Mann-Whitney U-test between ACS patients and control patients (*P-value ACS*) and between stable patients and control patients (*P-value Stable CAD*).

Abbreviations: ACS= acute coronary syndrome; CAD = coronary artery disease; HGF= hepatocyte growth factor; IFN-γ= interferon gamma; IL=interleukin; M-CSF= macrophage colony-stimulating factor; MIP-1β= macrophage inflammatory protein 1 beta; PDGF-BB= platelet derived growth factor -BB; SCGF-β= stem cell growth factor beta; TNF-α= tumor necrosis factor alpha.

Supplementary Table S3. Cytokine concentration's Z-scores of cytokines with p-value <0.05 in the FACT cohort.

Cytokines	FACT				
	ACS Median (IQR)	Stable CAD Median (IQR)	Controls Median (IQR)	P-value ACS	P-value Stable CAD
IL-18	1.455 (-0.059–2.711)	1.159 (-0.256–2.643)	-0.144 (-0.587–0.549)	0.002	0.011
IL-9	0.394 (-0.434–1.562)	0.611 (0.167–1.230)	-0.031 (-0.485–0.660)	0.079	0.005
SCGF-β	0.328 (-0.604–1.305)	0.701 (-0.275–2.169)	-0.183 (-0.754–0.391)	0.150	0.025

Data are shown as medians and 25th-75th interquartile range (IQR). P-values are from Mann-Whitney U-test between ACS patients and control patients (*P-value ACS*) and between stable patients and control patients (*P-value Stable CAD*).

Abbreviations: ACS= acute coronary syndrome; CAD = coronary artery disease; IL=interleukin; SCGF-β= stem cell growth factor beta.

Supplementary Table S4. P-values and risk estimates from adjusted logistic regression models in both study cohorts compared to disease-free controls.

Cytokines	CAREBANK				FACT			
	ACS OR (95% CI)	P-value	Stable CAD OR (95% CI)	P-value	ACS OR (95% CI)	P-value	Stable CAD OR (95% CI)	P-value
IL-4	1.007 (0.585–4.137)	0.964	1.575 (1.170–2.113)	0.003	1.405 (0.559–3.531)	0.470	1.273 (0.611–2.650)	0.520
SCGF-β	0.717 (0.488–1.054)	0.090	0.514 (0.321–0.824)	0.006	1.973 (0.939–4.147)	0.073	1.679 (1.032–2.733)	0.037
IFN-γ	0.968 (0.692–1.355)	0.852	1.468 (1.098–1.962)	0.009	1.951 (0.728–5.226)	0.184	1.491 (0.696–3.197)	0.305
MCP-3	0.500 (0.208–1.200)	0.121	0.220 (0.071–0.682)	0.009	0.966 (0.631–1.480)	0.875	0.913 80.470–1.773)	0.788
TNF-α	1.575 (1.111–2.234)	0.011	1.290 (0.925–1.799)	0.134	1.928 (0.785–4.735)	0.152	2.068 (0.884–4.840)	0.094
Eotaxin	1.177 (0.805–1.722)	0.401	1.572 (1.096–2.255)	0.014	1.025 (0.569–1.846)	0.935	0.769 (0.373–1.584)	0.476
IL-17	1.009 (0.692–1.471)	0.963	1.419 (1.006–2.000)	0.046	3.847 (1.435–10.313)	0.007	2.668 (1.195–5.956)	0.017
IL-18	0.735 (0.483–1.119)	0.151	0.759 (0.513–1.124)	0.169	2.050 (1.271–3.307)	0.003	1.934 (1.177–3.178)	0.009

Abbreviations: ACS= acute coronary syndrome; CAD = coronary artery disease; CI = confidence interval; IFN-γ= interferon gamma; IL=interleukin; MCP-3= MCP= monocyte-chemotactic protein 3; OR = odds ratio; SCGF-β= stem cell growth factor beta; TNF-α= tumor necrosis factor alpha.
